# Fine‐tuning ethanol oxidation pathway enzymes and cofactor PQQ coordinates the conflict between fitness and acetic acid production by *Acetobacter pasteurianus*

**DOI:** 10.1111/1751-7915.13703

**Published:** 2020-11-11

**Authors:** Ling Gao, Xiaodan Wu, Xiaole Xia, Zhengyu Jin

**Affiliations:** ^1^ State Key Laboratory of Food Science and Technology School of Food Science and Technology Jiangnan University Wuxi China; ^2^ State Key Laboratory of Biobased Material and Green Papermaking Qilu University of Technology Shandong Academy of Sciences Jinan China; ^3^ The Key Laboratory of Industrial Biotechnology Ministry of Education School of Biotechnology Jiangnan University Wuxi China

## Abstract

The very high concentrations required for industrial production of free acetic acid create toxicity and low pH values, which usually conflict with the host cell growth, leading to a poor productivity. Achieving a balance between cell fitness and product synthesis is the key challenge to improving acetic acid production efficiency in metabolic engineering. Here, we show that the synergistic regulation of alcohol/aldehyde dehydrogenase expression and cofactor PQQ level could not only efficiently relieve conflict between increased acetic acid production and compromised cell fitness, but also greatly enhance acetic acid tolerance of *Acetobacter pasteurianus* to a high initial concentration (3% v/v) of acetic acid. Combinatorial expression of *adhA* and *pqqABCDE* greatly shortens the duration of starting‐up process from 116 to 99 h, leading to a yield of 69 g l^‐1^ acetic acid in semi‐continuous fermentation. As a final result, average acetic acid productivity has been raised to 0.99 g l^‐1^ h^‐1^, which was 32% higher than the parental *A. pasteurianus*. This study is of great significance for decreasing cost of semi‐continuous fermentation for producing high‐strength acetic acid industrially. We envisioned that this strategy will be useful for production of many other desired organic acids, especially those involving cofactor reactions.

## Graphical abstract

Cofactor engineering is employed by manipulating ethanol respiration chain dehydrogenase module expression, cofactor PQQ biosynthesis module and various combinations of two modules to further improve acetic acid productivity.The synergistic regulation of alcohol/aldehyde dehydrogenase expression and cofactor PQQ level could not only efficiently relieve conflict between increased acetic acid production and compromised cell fitness, but also greatly enhance acetic acid tolerance of Acetobacter pasteurianus to a high initial concentration (3% v/v) of acetic acid.

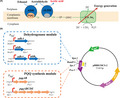

## Introduction

Metabolic engineering manipulates the cellular metabolism of microbes to maximize metabolic flux towards a desired product formation pathway (Smanski, *et al*., 2016; He, *et al*., 2017). Since cellular metabolism is strictly regulated to produce metabolites required for cell growth, overexpression of endogenous/heterologous pathways often leads to competition between cell growth and the desired product formation (Wu, *et al*., 2016; Soma, *et al*., 2017; Tsoi, *et al*., 2018). In particular, when the heterologous proteins or pathway intermediates/products are toxic to host, overproduction of toxic proteins or intermediates/products leads to growth retardation or adaptive responses that reduce productivity (Chubukov, *et al*., 2016; Wu, *et al*., 2016; Davy, *et al*., 2017). To obtain economically viable fermentation and further improvements in chemical production, it is essential to manage this trade‐off phenomenon.

**Fig. 1:**
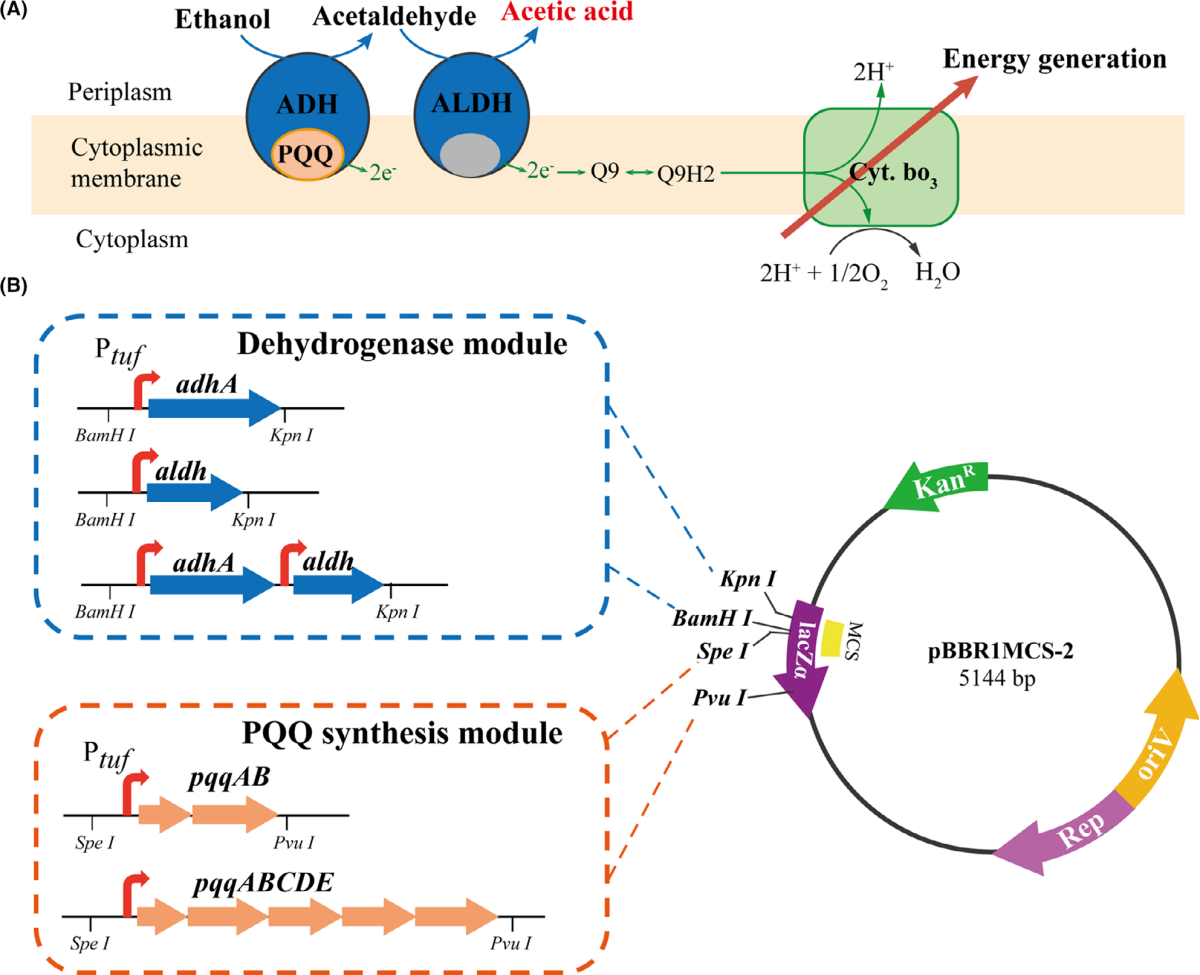
Engineering *A. pasteurianus* for enhancement of acetic acid production. A. ethanol oxidation pathway is the main acetic acid and energy‐producing pathway by the two membrane‐bound enzymes: alcohol dehydrogenase (ADH) and (ALDH). The cofactor pyrroloquinoline quinone (PQQ) is required in this ethanol oxidation pathway as the mediator of electron transfer. B. Schematic diagram of the engineered *A. pasteurianus* with different combination of genetic constructs in this study. The key dehydrogenases were constructed in 3 ways: P*_tuf_*‐*adhA*, P*_tuf_*‐*aldh* and P*_tuf_*‐*adhA*‐*aldh*. PQQ biosynthesis is encoded by *pqq* gene cluster, which was constructed in 2 ways: P*_tuf_*‐*pqqAB* and P*tuf*‐*pqqABCDE*. These two modules were further combined differentially and cloned into the same broad‐host vector pBBR1MCS‐2, which led to 3*2 = 6 combinations in total.

Acetic acid is a weak organic acid that exerts a toxic effect on most microorganisms at concentrations as low as 0.5%(w/w), and it usually serves as an effective food preservative to prevent the growth of pathogenic and spoilage organisms in fermented foods (Gullo, *et al*., 2014). Acetic acid bacteria (AAB), especially the *Acetobacter* and *Komagataeibacter* species, are used industrially as acetic acid producers, owing to their high resistance to acetic acid. Acetic acid fermentation is a typical case in which product toxicity conflicts with cell growth. On the one hand, acetic acid fermentation is performed by membrane‐bound alcohol dehydrogenase (ADH) and aldehyde dehydrogenase (ALDH) in acetic acid bacteria (AAB) (Lynch, *et al*., 2019). As shown in Fig. 1A, membrane‐bound ADH and ALDH not only catalyse the conversion of ethanol substrate to acetic acid but also couple the respiration chain through reduction of ubiquinone to ubiquinol. The electrons released by oxidation from ethanol substrate flow directly into electron transport chain to reduce oxygen to water, which is coupled to the production of ATP (Matsushita, *et al*., 2016). Thus, acetic acid accumulates outside the cells with production of energy inside the cells, and the ethanol is primarily used as an energy source to support cell growth (Qi, *et al*., 2014a; Zheng, *et al*., 2017). On the other hand, rapid acetic acid accumulation generally causes severe acid stress, and this stress could harm cell fitness and inhibit the initiation of cell cultures by decreasing intracellular pH and disturbing energy metabolism (Trček, *et al*., 2015; Xia, *et al*., 2015). Semi‐continuous fermentation mode is reported to be the most advantageous for high‐titre acetic acid production. An initial acetic acid concentration of 35–40 g l^‐1^ is usually retained for semi‐continuous fermentation, until the final acetic acid content increases to 70–100 g l^‐1^ (Qi, *et al*., 2014a; Krusong, *et al*., 2015). For example, Krusong, *et al*. (2014) assessed the effect of a stepwise increment of initial acetic acid concentration in fermentation by high acid‐tolerant strain of *A. aceti* WK. Although their total acid concentration was even increased to 100 g l^‐1^, the average productivity still is very low. Growth retardation caused by high concentration acetic acid is a bottleneck that reduced productivity of semi‐continuous fermentation. Therefore, exploring the trade‐off relationship and further obtaining an optimum balance between improved acetic acid production and cell fitness is a vital step in the strain engineering process.

Previous studies on strain improvement were mostly focused on enhancing ethanol oxidation to increase acetic acid production, using measures such as overexpressing membrane‐bound ADH or ALDH (Fukaya, *et al*., 1989; Wu, *et al*., 2017). Several fermentation engineering optimization strategies showed that enhancing energy generation by facilitating the electron transfer and oxidative phosphorylation could not only promote cell growth but also increase acetic acid production, through actions such as supplementing precursors of the electron transfer carrier coenzyme Q and oxygen electron acceptor (Qi, *et al*., 2014b; Xia, *et al*., 2015). But, these precursors, such as haeme, coenzyme Q, isopentenyl alcohol and β‐hydroxybenzoic acid, are very expensive, which greatly increase the cost of product acetic acid. In fact, engineering cofactor by manipulating availability of cofactors such as NADH, NADPH and ATP proved to be an effective strategy for coordinating material and energy needs, and it substantially improved the yields of target products (Wang, *et al*., 2017). NADH or ATP‐driven systems (Lan and Liao, 2012; Ji, *et al*., 2013) have been devised as a potential solution for dilemmas related to cell growth and target chemical production. Pyrroloquinoline quinone (PQQ) has been determined to be another important cofactor, after NADH and NADPH, serving as a redox cofactor for a large number of dehydrogenases known as quinoproteins (Misra, *et al*., 2012). It has been shown to improve the adaptability of microorganisms to extreme environments, to factors such as radiation, high acid concentrations and high temperatures (Choi, *et al*., 2008). Various levels of PQQ in *Gluconobacter oxydans* could be generated by overexpressing various parts of the PQQ biosynthesis gene clusters, such as *pqqA*, *pqqABCDE* and *pqqABCDEN* (Du, *et al*., 2013; Gao, *et al*., 2014; Wang, *et al*., 2016). Moreover, the balanced coexpression of sorbose/sorbosone dehydrogenase and cofactor PQQ could increase the 2‐keto‐L‐gulonic acid production and maintain specific growth rate of *Ketogulonigenium vulgare–Bacillus cereus* consortium (Du, *et al*., 2013; Gao, *et al*., 2014). In *A. pasteurainus*, PQQ, served as cofactor of membrane‐bound ADH, is involved in electron transfer of ethanol respiration chain (Matsutani and Yakushi, 2018), but it remains vague how the growth rate and acetic acid production would be affected by PQQ level generation. And the manipulation of expression level of PQQ synthases was also not examined in *A. pasteurainus*. The availability of cofactor PQQ and its balance in relation to expression of ethanol oxidation pathway enzymes have also generally been neglected.

Therefore, in this study, we manipulated the expression of ethanol respiration chain‐dehydrogenase module, cofactor PQQ biosynthesis module and various combinations of the two modules to coordinate the conflict between cell fitness and acetic acid production. The concentration of PQQ, cell fitness and acetic acid production were detected in wild‐type *A. pasteurianus* and engineered strains in fermentation medium supplemented with 0, 1% (v/v) and 3% (v/v) initial acetic acid concentrations. Fine‐tuning the balance between ethanol oxidation pathway enzymes and PQQ regeneration level not only relieves the conflicts between fitness and acetic acid production but also enables *A. pasteurianus* cells to grow in environments with very high acetic acid concentrations.

## Results

### Effect of overexpressing membrane‐bound ADH/ALDH on A. pasteurianus fitness and acetic acid biosynthesis

**Fig. 2:**
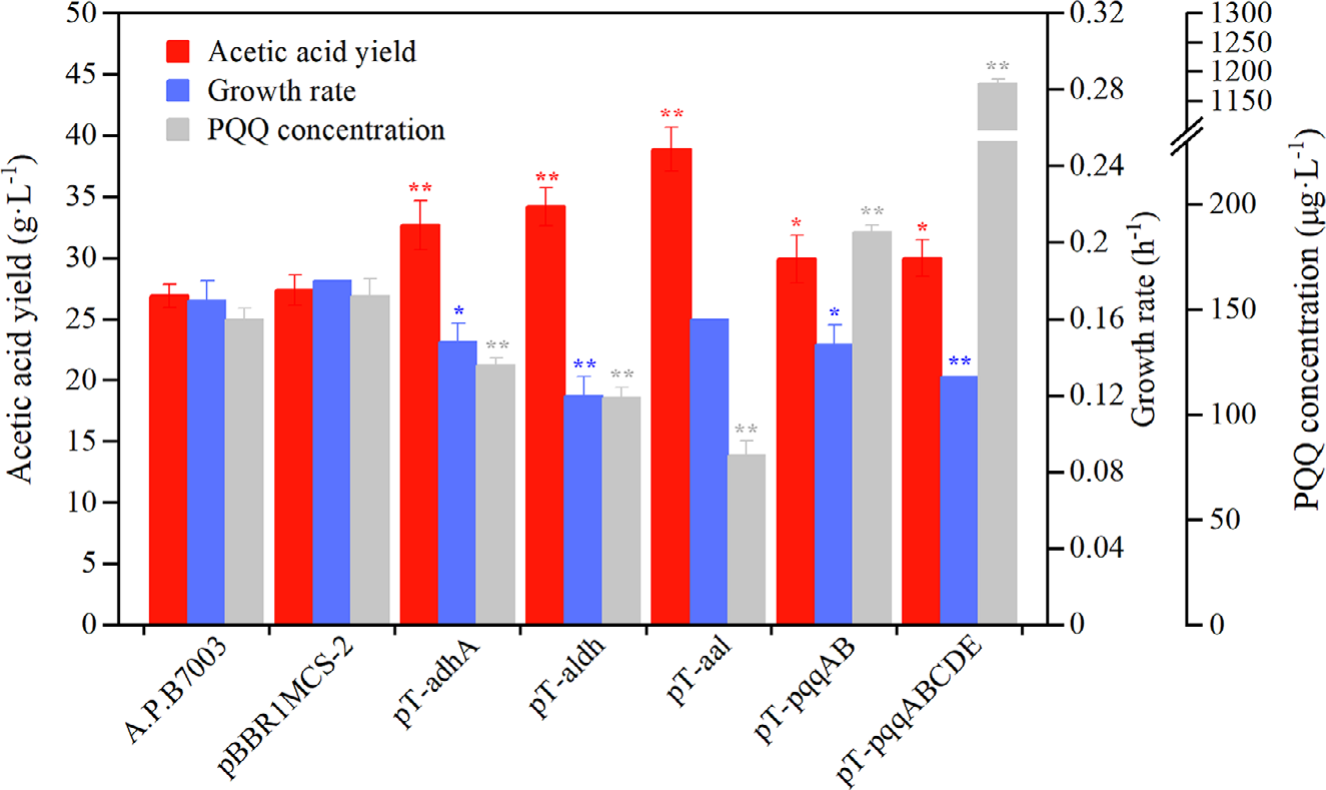
Effects of overexpression of dehydrogenase module and cofactor PQQ module on*A. pasteurianus*. Error bars represent the standard deviation of three biological replicates. Comparison was performed with wild‐type*A. pasteurianus*B7003 (* and **P < 0.05 and 0.01 respectively).

Our previous study showed that promoter P*_tuf_* is 1.8‐fold stronger than P*_adhA_* in the overexpression of green fluorescent protein (GFP) in *A. pasteurianus* (see Fig. S2). To enhance acetic acid production, we overexpressed *adhA* and *aldh* genes with the strong promoter P*_tuf_* in a broad‐host vector pBBR1MCS‐2 in *A. pasteurianus*. The growth rate (*µ*) was used as a proxy for strain’s fitness (Brauer, *et al*., 2008; Bershtein, *et al*., 2015). As shown in Fig. 2 and Table S2, the wild‐type *A. pasteurianus* B7003 produced 26.89 ± 0.93 g l^‐1^ acetic acid after 24 h of incubation (*µ* = 0.17 ± 0.01 h^‐1^, W = 1.00). *A. pasteurianus* strain with control plasmid showed the same acetic acid yield and growth rate as wild type, which indicated that the exogenous blank plasmid did not place a metabolic burden on cells. As expected, three mutants with *adhA*, *aldh* and *adhA*‐*aldh* overexpression all showed increases in acetic acid yields (Fig. 2). In particular, the coexpression sample (*A. pasteurianus/*pT‐aal) displayed a highest level of acetic acid, at 38.86 ± 1.80 g l^‐1^ and simultaneously kept similar growth to wild type. However, the mutants *A. pasteurianus*/pT‐adhA and *A. pasteurianus*/pT‐aldh both showed a moderate drop in cell growth (*P *< 0.05), which indicated that their fitness was threatened.

### Effect of endogenous PQQ biosynthesis pathway overexpression

PQQ serves as cofactor of membrane‐bound ADH/ALDH that catalyses redox reaction (and electron transfer) from ethanol to acetic acid (Sengun, *et al*., 2017). The concentrations of PQQ were analysed in wild‐type and engineered strains. As shown in Fig. 2 and Table S2, PQQ level was decreased in all mutants with dehydrogenase module overexpression (*P* < 0.01). In particular, PQQ level in coexpression strain *A. pasteurianus*/pT‐aal was seriously decreased to 80.75 ± 6.82 μg l^−1^ at approximately 24 h (wild type: 145.25 ± 5.42 μg l^−1^). We hypothesized that the imbalanced concentration between PQQ and dehydrogenases might be a bottleneck that inhibited further improvement of acetic acid production.

To explore whether PQQ could enhance acetic acid production in engineered strains, we first determined the effects of *pqqAB* and *pqqABCDE* gene overexpression on PQQ regeneration, cell growth and acetic acid production in *A. pasteurianus* (Fig. 2 and Table S2). As expected, mutant strain *A. pasteurianus*/pT‐pqqAB enhanced PQQ level by 29% (187.00 ± 2.97 µg l^‐1^) and *A. pasteurianus* mutant harbouring a plasmid bearing the complete *pqqABCDE* cluster (pT‐pqqABCDE) achieved an eightfold higher level of PQQ biosynthesis (1181.25 ± 7.36 µg l^‐1^) than wild type (145.3 ± 5.42 µg l^‐1^). This result confirmed that overexpression of *pqqAB* and *pqqABCDE* in *A. pasteurianus* enabled differential increase in PQQ level. Moreover, *A. pasteurianus* mutants with overexpression of *pqqAB* and *pqqABCDE* both increased acetic acid yield by 12% (30.00 ± 1.50 g l^‐1^) than wild type, which suggested that increasing PQQ regeneration has a positive effect on acetic acid production. However, the growth rates of mutants (*A. pasteurianus*/pT‐pqqAB and *A. pasteurianus*/pT‐pqqABCDE) were lower than that of wild type (*P* < 0.05). And strain *A. pasteurianus*/pT‐pqqABCDE with a high PQQ level displayed a slower growth rate than *A. pasteurianus*/pT‐pqqAB (Fig. 2). These results indicated that overexpression of the endogenous PQQ biosynthesis pathway genes also resulted in a trade‐off between improved acetic acid production and compromised cell fitness.

### Effect of co‐overexpressing membrane‐bound ADH/ALDH and PQQ biosynthesis pathway

**Fig. 3:**
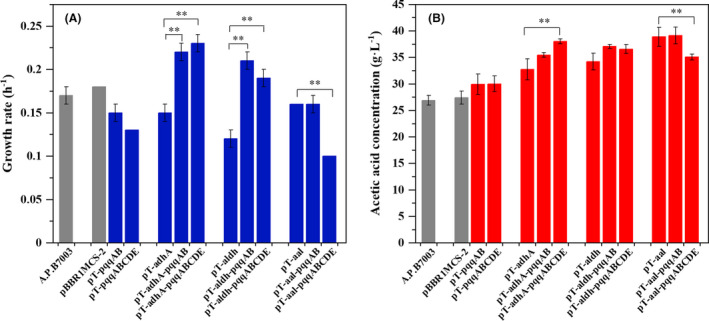
Growth rate (A) and acetic acid production (B) of the wild‐type*A. pasteurianus*and all engineered strains in the fermentation medium supplemented without initial acetic acid. The grey columns represented wild‐strain*A. pasteurianus*and*A. pasteurianus*/pBBR1MCS‐2. The blue and red columns represented growth rate and acetic acid production respectively. Error bars showed the standard deviation of three biological replicates, **P < 0.01.

Subsequently, to investigate the balancing relationship between dehydrogenase module and cofactor PQQ module on cell fitness and acetic acid production, six expression patterns in the combined genes from acetic acid biosynthesis and PQQ biosynthesis pathways were constructed and determined in *A. pasteurianus* (as shown in Fig. 1 B). The growth rate and acetic acid production are summarized in Fig. 3 and Table S2. In comparison with *A. pasteurianus*/pT‐adhA, coexpression of *adhA* with PQQ biosynthesis genes, which increased growth rates by 49% and 56% in the *A. pasteurianus*/pT‐adhA‐pqqAB and *A. pasteurianus*/pT‐adhA‐pqqABCDE strains, simultaneously increased acetic acid yields from 32.71 ± 1.99 to 35.44 ± 0.44 and 38.01 ± 0.45 g l^‐1^ (Fig. 3). Similarly, coexpression of *aldh* and *pqqAB*/*pqqABCDE* both had a positive effect on growth rate and increased acetic acid yields from 34.20 ± 1.57 to 37.05 ± 0.37 g l^‐1^ (Table S2). These results indicated that the synergistic improvement of membrane‐bound ADH/ALDH and cofactor PQQ modules could relieve conflict between acetic acid production and compromised cell growth, thereby maintaining acetic acid production and simultaneously improving cell fitness. However, the coexpression of *pqqAB* had little effect on growth rate and acetic acid yield in strain *A. pasteurianus*/pT‐aal, leading to a highest production of acetic acid (39.14 ± 1.58 g l^‐1^). The expression of *pqqABCDE* in strain *A. pasteurianus*/pT‐aal led to 38% decrease of growth rate and marginally decreased acetic acid from 38.86 ± 1.80 to 35.05 ± 0.57 g l^‐1^ (Fig. 3), suggesting that intracellular PQQ level achieved by *pqqABCDE* was not optimal level for improving acetic acid yield in strain *A. pasteurianus*/pT‐aal.

### Effect of initial acetic acid on fitness and acetic acid fermentation by recombinants

The high acetic acid tolerance of *A. pasteurianus* is important for semi‐continuous fermentation for producing high‐strength acetic acid industrially. It is commonly accepted that good determination and control of the starting‐up process will determine productivity of a semi‐continuous fermentation process (Charles, *et al*., 2009). In previous work, we proposed a two‐stage starting‐up protocol to achieve high acid production, in which 10 g l^‐1^ of initial acetic acid was used to promote acetic acid production, and then. acetic acid from 35 to 40 g l^‐1^ was retained for the second starting‐up fermentation, until the final acetic acid content increased to approximately 70 g l^‐1^ (Xia, *et al*., 2015). However, a high initial concentration of acetic acid at 35–40 g l^‐1^ often results in a long lag phase during the second starting‐up process.

**Fig. 4:**
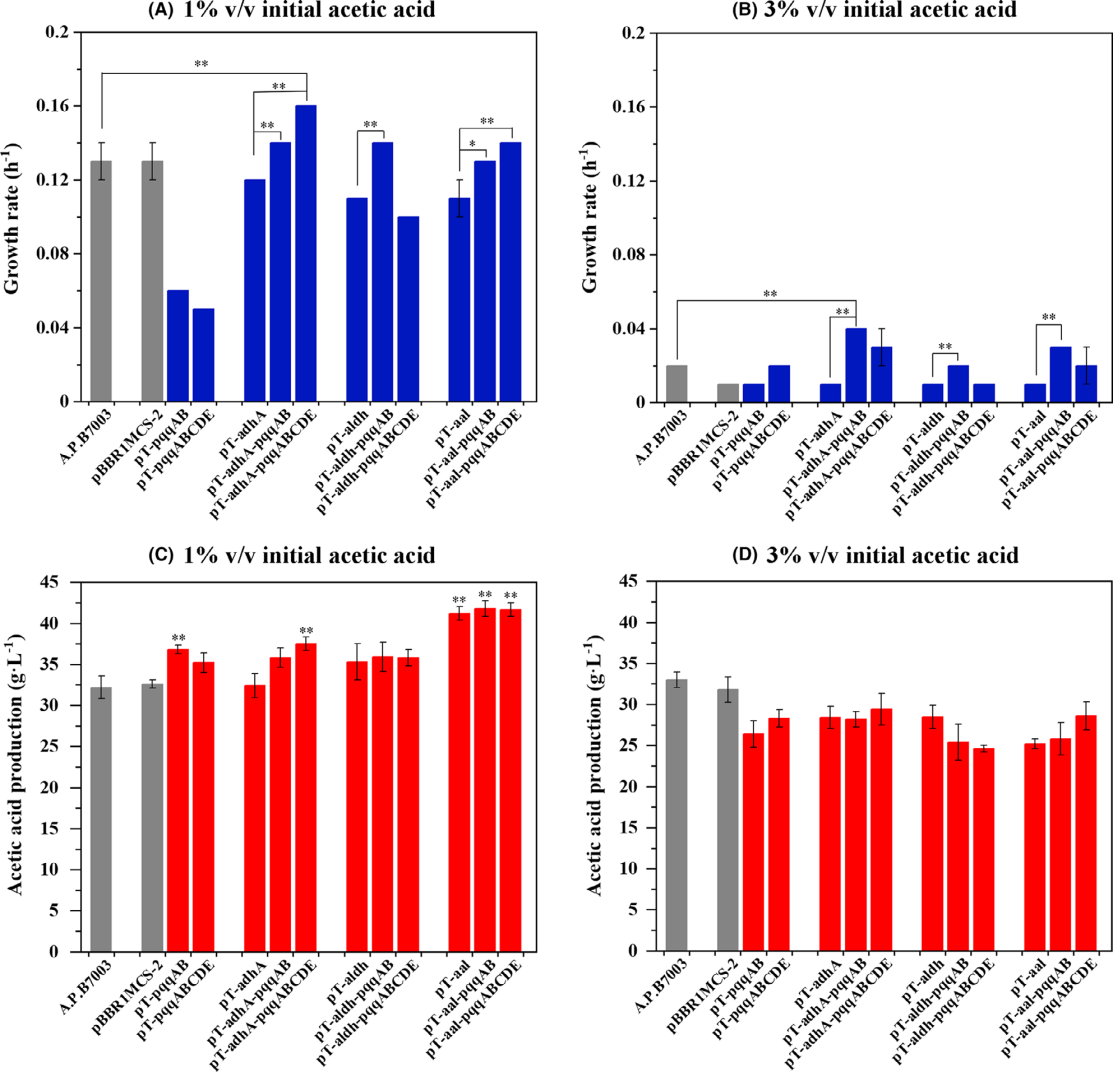
Growth rate (A, B) and acetic acid production (C, D) of the wild‐type*A. pasteurianus*and all engineered strains in fermentation medium supplemented with 1% (v/v) and 3% (v/v) initial acetic acid. The concentration of acetic acid has been subtracted from initial acetic acid. The grey columns represented wild‐strain*A. pasteurianus*and*A. pasteurianus*/pBBR1MCS‐2. The blue and red columns represented growth rate and acetic acid production respectively. Error bars showed the standard deviation of three biological replicates, **P < 0.01 when compared to wild‐type*A. pasteurianus*.

In our study, we simulated the acidic environment over the entire starting‐up stage to determine acetic acid production and tolerance of engineered strains. Concentrations of 1% (v/v) and 3% (v/v) acetic acid were added to fermentation medium from the beginning. As expected, acetic acid inhibited cell growth in a concentration‐dependent manner, including that of all the overexpression strains (Fig. 3A, Fig. 4A and B). Similar to reduced growth rate, PQQ regeneration level also decreased with the increased initial acetic acid concentration (Table S2, S3 and S4). Differently, acetic acid production of all strains reached a maximum value in the presence of 1% (v/v) initial acetic acid (Figs 3 and 4). This result is consistent with previous study in which a low concentration of initial acetic acid had a positive effect on acetic acid production (Wang, *et al*., 2015). Among all strains, *A. pasteurianus* strains harbouring *adhA‐aldh* genes (i.e. pT‐aal, pT‐aal‐pqqAB and pT‐aal‐pqqABCDE) produced the highest acetic acid, with a yield of 41.21 ± 0.83, 41.75 ± 0.95 and 41.69 ± 0.82 g l^‐1^, respectively, in the presence of 1% initial acetic acid (Table S3). Their productivities (1.61 ± 0.07, 1.65 ± 0.09 and 1.67 ± 0.04 g l^‐1^ h^‐1^) were also significantly higher than from *A. pasteurianus* JST‐S/pBBR‐adhA‐adhB (Wu, *et al*., 2017) and *A. pasteurianus*/pMV24‐*uvrA* (Zheng, *et al*., 2018), under the condition of shake flask with 1% initial acetic acid at 30° (Table 1). However, in the presence of 3% (v/v) initial acetic acid, acetic acid production of all engineered strains showed a significant drop compared to wild type (Fig. 4 D). This decrease was most likely caused by metabolic flux change induced by the high initial concentration of acetic acid. The TCA cycle, which assimilates intracellular acetic acid, was upregulated to produce more energy in response to high acetic acid stress, which led to decreased acetic acid production in all the recombinants. Interestingly, fine tuning PQQ regeneration level by PQQ biosynthesis genes could also effectively improve growth rate of engineered strains with dehydrogenase expression module (i.e. *A. pasteurianus*/pT‐adhA, *A. pasteurianus*/pT‐aldh and *A. pasteurianus*/pT‐aal) in the presence of initial acetic acid (Fig. 4A and B). In particular, the growth rates of engineered strains *A. pasteurianus*/pT‐adhA‐pqqABCDE and *A. pasteurianus*/pT‐adhA‐pqqAB, respectively, showed a significant increased in presence of 1% (v/v) or 3% (v/v) acetic acid, which indicated that they are likely to be more resistant to acetic acid than wild‐type. Thus, a spot assay experiment was performed to detect two strains’ acetic acid tolerance (Fig. S3). Obviously, above two strains grew better than wild type on fermentation medium plates containing 2 and 3 g l^‐1^ acetic acid. These results further demonstrated that balanced relationship between dehydrogenase and cofactors played an important role on cell growth and tolerance in *A. pasteurianus*.

**Table 1:** Comparison of acetic acid production in engineered *A. pasteurianus* and *A. aceti* strains.

Microorganism hosts	Fermentation temperature (℃)	Total acid (g l^‒1^)	Productivity (g l h^‒1^)	Carbon source	Fermentation mode	**References**
*A. pasteurianus* JST‐S/pBBR‐adhA‐adhB	30	61.4	0.71	Ethanol	Shake flask	(Wu, *et al*., 2017)
*A. pasteurianus*/pMV24‐*uvrA*	30	85	1.57	Ethanol	Shake flask	(Zheng, *et al*., 2018)
*A. aceti* 10‐8S2/pACO300	30	105	0.76	Ethanol	Batch	(Nakano, *et al*., 2004)
*A. aceti* 10‐8S2/pABC101	30	111.7	0.89	Ethanol	Shake flask	(Nakano, *et al*., 2006)
*A. acetic WK*	30	100	9.57 g l^‒1^ day^‒1^	Ethanol	Semi‐continuous (nine repeat batches)	(Krusong, *et al*., 2015)
*A. pasteurainus* UMCC 2951	30	80	6 g l^‒1^ day^‒1^	Ethanol	Semi‐continuous (five repeat batches)	(Ruttipron, *et al*., 2020)
*A. pasteurainus* CV01	38	100	1.08	Ethanol	Semi‐continuous (one repeat batch)	(Majid, *et al*., 2016)
*A. pasteurianus* CICIM B7003‐02	30	93.1	1.83	Ethanol	Semi‐continuous (discharge/charge ratio optimization and aeration strategy, four repeat batches)	(Qi, *et al*., 2014a)
*A. pasteurainus*/pT‐adhA‐pqqABCDE	30	68.8	0.99	Ethanol	Semi‐continuous (two repeat batches)	This study

### Semi‐continuous fermentation of engineered strains for high‐strength acetic acid production

**Fig. 5:**
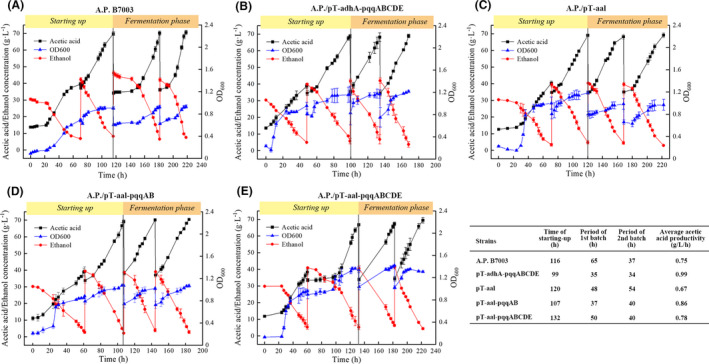
Semi‐continuous fermentation characteristics of original strain*A. pasteurianus*B7003 (A) and engineered strain*A. pasteurianus*/pT‐adhA‐pqqABCDE (B),*A. pasteurianus*/pT‐aal (C),*A. pasteurianus*/pT‐aal‐pqqAB (D) and*A. pasteurianus*/pT‐aal‐pqqABCDE (E). Error bars showed the standard deviation of three biological replicates.

Taking consideration of acetic acid production and growth rate, engineered strains *A. pasteurianus*/pT‐adhA‐pqqABCDE, *A. pasteurianus*/pT‐aal, *A. pasteurianus*/pT‐aal‐pqqAB and *A. pasteurianus*/pT‐aal‐pqqABCDE were presumed to be a good starter culture candidate for high‐strength acetic acid production. We further performed semi‐continuous culture experiments of these four engineered strains and original strain *A. pasteurianus* B7003 in a well‐controlled 7.5 L fermenter with a working volume of 4 L to produce high‐strength acetic acid (Fig. 5). For *A. pasteurianus* B7003, it took 116 h to complete starting‐up, leading to a yield of 70 g l^‐1^ acetic acid (Fig. 5A). Subsequently, two repeated batches of fermentation were performed. The period of first fermentation batch was 65 and 37 h for second batch. As a result, the average acetic acid productivity of whole process is 0.75 g l^‐1^ h^‐1^ in original strain *A. pasteurianus* B7003. In comparison, semi‐continuous culture of the engineered strain *A. pasteurianus*/pT‐adhA‐pqqABCDE displayed the best performance, which resulted in 99 h of staring‐up with average productivity of 0.99 g l^‐1^ h^‐1^. The period of each fermentation batch was 34–35 h (Fig. 5 B). Obviously, cell growth and acetic acid accumulation of engineered strain *A. pasteurianus*/pT‐adhA‐pqqABCDE were faster than *A. pasteurianus* B7003 in the starting‐up phase due to the synergistic expression of ADH and cofactor PQQ. The result is also better than from *A. acetic WK* (Krusong, *et al*., 2015) and *A. pasteurainus* UMCC 2951 (Ruttipron, *et al*., 2020), under the condition of semi‐continuous fermentation (Table 1). Similar to flask experiments, the performance of strain *A. pasteurianus*/pT‐aal with overexpression of PQQ both are superior to *A. pasteurianus*/pT‐aal, owing to their better growth on fermentation medium with initial acetic acid. In general, higher acetic acid productivity and cell fitness of *A. pasteurianus*/pT‐adhA‐pqqABCDE, *A. pasteurianus*/pT‐aal‐pqqAB and *A. pasteurianus*/pT‐aal‐pqqABCDE demonstrated the effectiveness of strategy of balanced coexpressed pathway enzymes and cofactor.

## Discussion

The redox cofactor PQQ is a member of secondary metabolites annotated as ribosomally synthesized and post‐translationally modified peptides and acts as a prosthetic group of alcohol and/or sugar dehydrogenases (Koehn, *et al*., 2019). The presence of this molecule has been shown to enhance cell growth rate (Ke, *et al*., 2019). Gene knockout and bioinformatics studies have identified that PQQ biosynthesis is accomplished by the gene products of a specific *pqq* operon (i.e. *pqqA*‐*E*), but a detail account of PQQ biosynthesis has remained unresolved (Holscher and Gorisch, 2006; Shen *et al*., 2012). Several recombinant strains have been developed to enhance PQQ production in *G. oxydans* by overexpression of each individual genes, and the PQQ levels followed the order: *pqqB *> *pqqA*>*pqqD *> *pqqC*>*pqqE* (Wang, *et al*., 2016). It has been reported that PQQ backbone (glutamate and tyrosine) is probably derived from PqqA peptide (Goosen, *et al*., 1992). Supplementation of amino acids involved in the biosynthesis of PQQ precursor peptide PqqA can effectively increase accumulation of PQQ in cells (Ke, *et al*., 2019). The PqqB protein takes part in transporting PQQ across the membrane and produces quinone moiety of the mature PQQ cofactor (Velterop, *et al*., 1995; Koehn, *et al*., 2019). The orthoquinone structure in PQQ is directly responsible for oxidoreduction, essential for the primary oxidation step of non‐phosphorylated substrates, such as alcohols, aldehydes or aldoses (Minenosuke and Toshiharu, 2018). PqqC catalyses the final step in PQQ formation (Magnusson, *et al*., 2004). PqqD as a novel peptide chaperone forms a ternary complex with the radical S‐adenosylmethionine protein PqqE (Latham, *et al*., 2015). PqqE catalyses de novo carbon–carbon cross‐linking within a peptide substrate PqqA in the presence of the peptide chaperone PqqD (Barr, *et al*., 2016). In our study, considering that ethanol oxidation occurs on cytoplasmic membrane, part of genes in *pqq* operon (i.e. *pqqAB*) and a full‐length *pqqABCDE* cluster were independently overexpressed to determine their impacts on PQQ biosynthesis and acetic acid production. Similar to previous report, overexpression of part of genes in PQQ biosynthetic gene cluster also can promote PQQ regeneration (Wang, *et al*., 2016), and the PQQ production followed order: *pqqAB < pqqABCDE* (Fig. 2). Although PQQ levels in *A. pasteurianus* were found to have a positive correlation with the conversion efficiency of ethanol to acetic acid, direct recombinant expression of PQQ biosynthetic gene cluster probably competes with precursor amino acids and energy required for cell growth, resulting in poor cell growth (Fig. 2).

In Asian countries (i.e. China and Japan), *A. pasteurianus* is commonly used for the industrial production of acetic acid, which could resist up to 6%(v/v) acetic acid. However, in European countries (i.e. Germany), *Komagataeibacter europaeus* is the main species used for the same purpose, which has been reported to exhibit 10% (v/v) acetic acid tolerance (Trcek, *et al*., 2006). Genomic analysis showed that there were significant intergeneric differences in the number of genes encoding PQQ‐ADH; *K. europaeus 5P3* contained up to 6 copies of PQQ‐ADH gene, while *A. pasteurianus* harboured only two copies (Wang, *et al*., 2015; Xia, *et al*., 2017). Our results demonstrated that increasing the number of copies of membrane‐bound ADH genes indeed enable *A. pasteurianus* stronger acetic acid production (Fig. 2). However, cell growth rate in strain *A. pasteurianus*/pT‐adhA slowed down comparing with original strain, which is consistent with previous reports in *A. pasteurianus* JST‐S (Wu, *et al*., 2017). Further study suggested that the synergistic regulation of cofactor PQQ and dehydrogenase plays an important role in coordinating conflict between acetic acid production and compromised growth rate. Especially in the semi‐continuous fermentation process, proper PQQ regeneration effectively shorted the lag phase of engineered strain *A. pasteurianus*/pT‐aal, leading to a higher productivity (Fig. 5). Cofactor PQQ generation, as an electron carrier, on one hand, accelerates the electron transfer in ethanol respiration chain, thus ensuring energy supply needed for cell growth and resistance to acetic acid stress. On the other hand, it may directly improve the catalytic activity and acetic acid stability of PQQ‐ADH that enables *A. pasteurianus* to grow and stay metabolically active at extremely high concentrations of acetic acid (Trcek, *et al*., 2006). In present study, *A. pasteurianus* strains harbouring *adhA* as the acetic acid biosynthesis module (i.e. pT‐adhA, pT‐adhA‐pqqAB and pT‐adhA‐pqqABCDE) displayed an increasing growth rate and increasing acetic acid production with the increasing PQQ level in presence of 0 or 1%(v/v) initial acetic acid (Figs 3 and 4). This result suggested that cofactor PQQ levels were positively correlated to PQQ‐ADH activity. Over the past few years, the cofactor of membrane‐bound ALDH remains unclear in acetic acid bacteria. Molybdopterin is presumed to be the putative prosthetic group of membrane‐bound ALDH in *K. europaeus* (Thurner, *et al*., 1997), whereas in *Ga. diazotrophicus*, PQQ is considered the prosthetic group (Gomez‐Manzo, *et al*., 2010). Different to co‐overexpression of ADH and PQQ, *A. pasteurianus*/pT‐aldh‐pqqABCDE with more PQQ level displayed a slower growth rate than *A. pasteurianus*/pT‐aldh‐pqqAB and they produced similar amounts of acetic acid (Figs 3 and 4). Thus, PQQ is probably not the cofactor of membrane‐bound ALDH in *A. pasteurianus*.

In recent years, with in‐depth study of the acetic acid‐tolerance mechanisms of AAB, many genes and proteins related to stress response and tolerance, such as membrane‐bound ADH (Wu, *et al*., 2017), nucleic acid repair enzyme UvrA (Zheng, *et al*., 2018), aconitase (Nakano, *et al*., 2004) and ABC transporter (Nakano, *et al*., 2006), have been successfully identified, facilitating the application of genetic improvement to improve acetic acid tolerance and product yield. A comparative of acetic acid productivity in engineered *A. pasteurianus* and *A. aceti* strains, was summarized in Table 1. Overall, the acidification rate of these engineered strains still stayed at flask level, and is lower than our work in presence of 1% initial acetic acid (Table S3). Under industrial conditions, although the high acid‐tolerant strain of *A. aceti* WK. and *A. pasteurianus* UMCC 2951 produced a total acid to 80–100 g l^‐1^, the period of each fermentation batch and acetification rate both were lower than our engineered strains (Fig. 5). Moreover, strains exhibit different degrees of resistance of acetic acid with the different fermentation phases of semi‐continuous. This resistance is affected by initiation of cell cultures, aeration rate, discharge/charge ratio, the number and modality of recursive cultivations in acetic acid media (Gullo, *et al*., 2014). For example, *A.pasteurianus* CICIM B7003‐02, an ultraviolet mutant from wild‐type strain *A.pasteurianus* CICIM B7003, produces a high acidity vinegar with an acetic acid concentration that reached up to 93.1 g l^‐1^ in the semi‐continuous mode via optimization of discharge/charge rate and aeration strategy (Qi, *et al*., 2014a). It should be noted that we just strengthened the ethanol respiratory chain from ethanol to acetic acid at the production stage. Further improvement in acetic acid resistance and productivity might be attained through optimizing process control.

In summary, this work represented the effectiveness of combining modular biological parts, in enhancing acetic acid production and tolerance, by constructing genes in ethanol oxidation pathway and in PQQ synthesis pathway as two modules. The engineered strain *A. pasteurianus*/pT‐adhA‐pqqABCDE with high cell fitness was obtained and serves as a good starter culture candidate for semi‐continuous fermentation, which is of great significance for decreasing cost of semi‐continuous fermentation for producing high‐strength acetic acid industrially. In the future, the engineering strategies can also be used to engineer cell factories for production of other organic acid.

## Experimental procedures

### Strains, media and culture conditions

*Acetobacter pasteurianus* CICIM B7003 isolated from a brewing factory (Hengshun Wantong Food Brewing Co., Ltd., Xuzhou, China) was used in this study. *Escherichia coli* JM109 used for general cloning was grown under routine conditions, on Luria–Bertani (LB) agar plates or in LB broth at 37°C. All the bacterial strains used in this study are shown in Table 2. The seed medium contained 10 g l^‐1^ glucose, 10 g l^‐1^ yeast extract and 3% (v/v) ethanol. The fermentation medium contained 10 g l^‐1^ glucose, 10 g l^‐1^ yeast extract, 0.6 g l^‐1^ KH_2_PO_4_, 0.4 g l^‐1^ MgSO_4_ and 4% (v/v) ethanol. When required, kanamycin (50 μg ml^‐1^ for *E. coli* or 25 μg ml^‐1^ for *A. pasteurianus*) was added to the culture medium. Cells from cryovials were incubated in 50 ml of seed medium in 250 ml Erlenmeyer flasks, and they were cultured at 30 °C for 24 h at 170 rpm. Fermentations were performed in fermentation medium at 30 °C at 220 rpm. Different initial concentrations of acetic acid were added to fermentation medium for detection of growth and production in *A. pasteurianus* and mutations.

**Table 2:** Bacterial strains and plasmids used in this study.

Name	Description	Reference or source
Strains		
*A. pasteurianus* CICIM B7003	Acetic acid production strain	Lab stock
*E. coli* JM109	endA1, recA1, gyrA96, thi, hsdR17 (rk^–^, mk^+^), relA1, supE44, Δ(lac‐proAB), [F´ traD36, proAB, laqIqZΔM15]..	Sangon Biotech
Plasmids		
pBBR1MCS‐2	A broad‐host vector, Kn^R^	Wang, *et al*. (2016)
pT‐adhA	Plasmid pBBR1MCS‐2 containing P*_tuf_*‐*adhA* from *A. pasteurianus*	This study
pT‐aldh	Plasmid pBBR1MCS‐2 containing P*_tuf_*‐*aldh* from *A. pasteurianus*	This study
pT‐aal	Plasmid pBBR1MCS‐2 containing P*_tuf_*‐a*dhA* and P_tuf_‐*aldh* from *A. pasteurianus*	This study
pT‐pqqAB	Plasmid pBBR1MCS‐2 containing P*_tuf_‐pqqAB* from *A. pasteurianus*	This study
pT‐pqqABCDE	Plasmid pBBR1MCS‐2 containing P*_tuf_‐pqqABCDE* from *A. pasteurianus*	This study
pT‐adhA‐pqqAB	Plasmid pBBR1MCS‐2 containing P*_tuf_*‐*adhA* and P*_tuf_‐pqqAB* from *A. pasteurianus*	This study
pT‐adhA‐pqqABCDE	Plasmid pBBR1MCS‐2 containing P*_tuf_*‐*adhA* and P*_tuf_‐pqqABCDE* from *A. pasteurianus*	This study
pT‐aldh‐pqqAB	Plasmid pBBR1MCS‐2 containing P*_tuf_*‐*aldh* and P*_tuf_‐pqqAB* from *A. pasteurianus*	This study
pT‐aldh‐pqqABCDE	Plasmid pBBR1MCS‐2 containing P*_tuf_*‐*aldh* and P*_tuf_‐pqqABCDE* from *A. pasteurianus*	This study
pT‐aal‐pqqAB	Plasmid pBBR1MCS‐2 containing P*_tuf_*‐a*dhA*, P*_tuf_*‐*aldh* and P*_tuf_‐pqqAB* from *A. pasteurianus*	This study
pT‐aal‐pqqABCDE	Plasmid pBBR1MCS‐2 containing P*_tuf_*‐a*dhA*, P*_tuf_*‐*aldh* and P*_tuf_‐pqqABCDE* from *A. pasteurianus*	This study

### Plasmid construction

All the plasmids used in this study are listed in Table 2. Plasmid construction and DNA manipulations were performed by following standard molecular biology techniques. All the primers used for PCR amplification are listed in Supplementary Table S1. Schematic diagrams of genetic constructs containing the enzyme genes from acetic acid biosynthesis pathway, PQQ biosynthetic genes and their various combinations are shown in Fig. 1.

The open reading frames (ORFs) of *adhA*, *aldh* and promoter of elongation factor TU (Gene ID: 8435080) as well as the *pqqAB* and *pqqABCDE* genes were amplified separately using genomic DNA of *A. pasteurianus*. The promoter of elongation factor TU was ligated with different *adhA*, *aldh*, *pqqAB* and *pqqABCDE* genes using SOE‐PCR. Subsequently, the resulting fragments P*_tuf_*‐*adhA* and P*_tuf_*‐*aldh* were inserted into *KpnI*‐*BamHI* sites of the pBBR1MCS‐2 plasmid using In‐Fusion Cloning, resulting in plasmids pT‐adhA, pT‐aldh and pT‐aal. The fragments P*_tuf_*‐*pqqAB* and P*_tuf_*‐*pqqABCDE* were digested and inserted at *SpeI*‐*PvuI* sites of the pBBR1MCS‐2 plasmid to produce pT‐pqqAB and pT‐ABCDE, and they were separately inserted into pT‐adhA, pT‐aldh or pT‐aal plasmids, generating six plasmids with different gene combinations (listed in Table 2). All the constructs were transformed into *A. pasteurianus* by electroporation (Zhang, *et al*., 2010).

### Analytical methods

The cell growth was monitored based on OD value at 600 nm using an EnSpire 2300 microplate reader (PerkinElmer, Waltham, MA, USA). The standard curve between OD_600_ and number of living bacteria (N) was obtained in *A. pasteurianus* and described in Fig. S1 (*N* = 10^3.1666*OD+7.0226^). The growth rates were determined from exponential growth phase using the three parameters in the fit of ln(N/N_0_) vs time curves proposed in Bershtein, *et al*. (2015). The relative fitness value (W) was calculated by finding ratio of the growth rate (mutant: ancestor) (Liu, *et al*., 2019). The total acid content was measured by titrating against 0.1 M NaOH with phenolphthalein as the pH indicator. The concentration of ethanol was determined by Hitachi HPLC system with an Hi‐Plex Ligand Exchange column (Agilent, 7.7 × 300 mm, 8 µm particle size). In this study, all experiments were performed in triplicate. The results were expressed as average values with a standard error.

### Measurement of PQQ

The PQQ concentration was measured using crude enzymes from *E. coli*/pET‐28a‐gcd containing apo‐glucose dehydrogenase with some modifications described in Wang, *et al*. (2016). In short, 500 μl of enzyme solution containing 250 μL of crude enzyme (approximately 0.4 mg protein), 250 μl of sample or a specific amount of PQQ standard and 10 mM MgSO_4_ in 50 mM phosphate buffer (pH 7.0) was incubated at 30 °C for 30 min. The reaction mixture was prepared by incubating 100 μl of enzyme solution, 0.20 M substrate glucose, 0.67 mM phenazine methosulfate (PMS) and 0.1 mM 2,6‐dichlorophenolindophenol (DCIP) in 1.0 ml of phosphate buffer pH 7.0 at 30 °C for 5 min. The absorbance changes in the reaction mixture were measured at 600 nm once the D‐glucose was added. The protein concentrations were measured using a Bradford Protein Assay kit (purchased from Sangon Biotech, Shanghai, China).

### Semi‐continuous fermentation

Semi‐continuous acetic acid fermentation was performed in a 7.5 l fermentor like our previous work (Qi, *et al*., 2014a). For starting‐up process, 3.16 l fermentation medium containing 10 g l^‐1^ acetic acid was poured into fermentor and mixed adequately with 0.3 l seeds. Aeration rate was set at 0.865 l min^‐1^ (0.25 vvm). When the residual ethanol concentration was below 5 g l^‐1^, 0.54 l fermentation medium with 260 g l^‐1^ ethanol was supplemented into fermentor to continue the starting‐up process. Simultaneously, aeration rate was set at 1.2 l min^‐1^ (0.3 vvm). Temperature was set at 30 °C for whole process. Starting‐up process was completed when the acetic acid content increased to about 70 g l^‐1^ with less than 5 g l^‐1^ residual ethanol. Subsequently, a new repeated batch was operated with discharging 43% (v/v) of total working volume (4 L) and then feeding the same volume of fresh fermentation medium containing 81.4 g l^‐1^ ethanol. Then, an acetification process was occurred as the previous one.

## Conflict of interest

None declared.

## Supporting information


**Fig. S1**. The relationship between the OD_600_ and the number of living bacteria (N, CFU/mL) in *A.pasteurianus* (N = 10^3.1666x+7.0226^).
**Fig. S2**. The whole cell fluorescence intensities of strains with P_adhA_‐GFP (A) and P_tuf_ ‐GFP (B). The promoter strength was defined as RFU/OD_600_ (relative fluorescence unit divided by the corresponding OD_600_)
**Fig. S3**. Acetic acid tolerance of wild‐type strain *A. pasteurianus* B7003, *A. pasteurianus*/pT‐adhA‐pqqAB and *A. pasteurianus*/pT‐adhA‐pqqABCDE. Cells were spotted in serial dilutions (diluted by a factor 10) and grown on fermentation medium agar plates amended with various concentrations of acetic acid.
**Tables S1**. PCR primers used for genetic constructs
**Table S2**. Summary of growth and production characteristics of *A. pasteurianus* B7003 and recombinants in fermentation medium (containing 4% (v/v) ethanol) without initial acetic acid.
**Table S3**. Summary of growth and production characteristics of *A. pasteurianus* B7003 and recombinants in fermentation medium (containing 4% (v/v) ethanol) supplemented with 1% (v/v) initial acetic acid. (The acetic acid yield has been subtracted from the initial acetic acid).
**Table S4**. Summary of growth and production characteristics of *A. pasteurianus* B7003 and recombinants in fermentation medium (containing 4% (v/v) ethanol) supplemented with 3% (v/v) initial acetic acid. (The acetic acid yield has been subtracted from the initial acetic acid).
