# Supplementary material for: Fine‐tuning ethanol oxidation pathway enzymes and cofactor PQQ coordinates the conflict between fitness and acetic acid production by Acetobacter pasteurianus
Source: Microb Biotechnol. 2020 Nov 11;14(2):643–55. doi: 10.1111/1751-7915.13703 (PMC7936290; doi:10.1111/1751-7915.13703)
Supplement: Supplementary file 1 — Fig. S1. The relationship between the OD600 and the number of living bacteria (N, CFU/mL) in A.pasteurianus (N = 103.1666x+7.0226). Fig. S2. The whole cell fluorescence intensities of strains with PadhA‐GFP (A) and Ptuf ‐GFP (B). The promoter strength was defined as RFU/OD600 (relative fluorescence unit divided by the corresponding OD600) Fig. S3. Acetic acid tolerance of wild‐type strain A. pasteurianus B7003, A. pasteurianus/pT‐adhA‐pqqAB and A. pasteurianus/pT‐adhA‐pqqABCDE. Cells were spotted in serial dilutions (diluted by a factor 10) and grown on fermentation medium agar plates amended with various concentrations of acetic acid. Tables S1. PCR primers used for genetic constructs Table S2. Summary of growth and production characteristics of A. pasteurianus B7003 and recombinants in fermentation medium (containing 4% (v/v) ethanol) without initial acetic acid. Table S3. Summary of growth and production characteristics of A. pasteurianus B7003 and recombinants in fermentation medium (containing 4% (v/v) ethanol) supplemented with 1% (v/v) initial acetic acid. (The acetic acid yield has been subtracted from the initial acetic acid). Table S4. Summary of growth and production characteristics of A. pasteurianus B7003 and recombinants in fermentation medium (containing 4% (v/v) ethanol) supplemented with 3% (v/v) initial acetic acid. (The acetic acid yield has been subtracted from the initial acetic acid). [file MBT2-14-643-s001.docx]

**Fine-tuning Ethanol Oxidation Pathway Enzymes and Cofactor PQQ Coordinates Conflict between Fitness and Acetic Acid production of *Acetobacter pasteurianus***

Ling Gao^1,3^, Xiaodan Wu^2^, Xiaole Xia^1,2^* and Zhengyu Jin^1*^

^1^State Key Laboratory of Food Science and Technology, School of Food Science and Technology, Jiangnan University, Wuxi, PR China

^2^The Key Laboratory of Industrial Biotechnology, Ministry of Education, School of Biotechnology, Jiangnan University, Wuxi, PR China

^3^State Key Laboratory of Biobased Material and Green Papermaking, Qilu University of Technology, Shandong Academy of Sciences, Jinan, PR China

***Correspondence**

Xiaole Xia, the Key Laboratory of Industrial Biotechnology, Ministry of Education, School of Biotechnology, Jiangnan University, 1800 Lihu Road, WuXi 214122, PR China

Tel./Fax: +86-510-85327270

E-mail: [xiaolexia@jiangnan.edu.cn](mailto:xiaolexia@jiangnan.edu.cn)

Zhengyu Jin, State Key Laboratory of Food Science and Technology, School of Food Science and Technology, Jiangnan University, 1800 Lihu Road, WuXi 214122, PR China

E-mail: zjin@jiangnan.edu.cn

**Table S1.** PCR primers used for genetic constructs

| **Primers** | **The sequence (5’ to 3’)** | **Restriction sites** |
| --- | --- | --- |
| TUF-F | GGATCCACGTTGCAAATATGCTTCA | BamHI |
| TUF-R | GCGCCTTTCCCGATCC |  |
| adh-F | atgacccgccccgcctcc |  |
| adh-R | GGTACCttaggggttaatgccaagtgtcggg | KpnI |
| Ta-soe-F | GGATCGGGAAAGGCGCatgacccgccccgcctcc |  |
| Ta-soe-R | GGAGGCGGGGCGGGTCATGCGCCTTTCCCGATCC |  |
| aldh-F | atgtcgtattatcttgaaatacc |  |
| aldh-R | GGTACCtcagtgcttttttgagtctgctc | KpnI |
| Tal-soe-F | GGATCGGGAAAGGCGCatgtcgtattatcttgaaatacc |  |
| Tal-soe-R | GGTATTTCAAGATAATACGACATGCGCCTTTCCCGATCC |  |
| TUF-speI-F | actagtACGTTGCAAATATGCTTCA | SpeI |
| pqqA-F | ATGGCTTGGACTGCACCAAAAGTAAC |  |
| pqqB-R | cgatcgTCATAGCAAGACTTTCATTCCATC | PvuI |
| pqqE-R | cgatcgTTACACTTTGGCGTTGCCATAGC | PvuI |
| TqABE-soe-F | GGATCGGGAAAGGCGCATGGCTTGGACTGCACCAAAAGTAAC |  |
| TqABE-soe-R | GTTACTTTTGGTGCAGTCCAAGCCATGCGCCTTTCCCGATCC |  |
| Tal-Fusion-F | ccgctctagaactagtggatccACGTTGCAAATATGCTTCA |  |
| Tal-Fusion-R | GAAGCATATTTGCAACGTtcagtgcttttttgagtctgctc |  |
| Ta-Fusion-F | gagcagactcaaaaaagcactgaACGTTGCAAATATGCTTCA |  |
| Ta-Fusion-R | agggaacaaaagctgggtaccttaggggttaatgccaagtgtcggg |  |

The underlined is the site of restriction endonuclease.

**Table S2.** Summary of growth and production characteristics of *A. pasteurianus* B7003 and recombinants in fermentation medium (containing 4% (v/v) ethanol) without initial acetic acid.

| Strains | OD_600_ | Growth rate  (h^-1^) | Relative fitness (W) | Acetic acid yield (g·L^-1^) | Acetic acid productivity  (g·L^-1^·h^-1^) | PQQ concentration (μg·L^-1^) |
| --- | --- | --- | --- | --- | --- | --- |
| *A. pasteurianus* B7003 | 0.63±0.03 | 0.17±0.01 | 1.00 | 26.89±0.93 | 1.01±0.02 | 145.25±5.42 |
| *A. pasteurianus*/pBBR1MCS-2 | 0.63±0.09 | 0.18±0.00 | 1.03 | 27.38±1.23 | 1.04±0.03 | 156.50±8.31 |
| **Overexpression of dehydrogenase module** | | | | | | |
| *A. pasteurianus*/pT-adhA | 0.59±0.04 | 0.15±0.01 | 0.86 | 32.71±1.99 | 1.23±0.09 | 123.50±3.55 |
| *A. pasteurianus*/pT-aldh | 0.44±0.04 | 0.12±0.01 | 0.71 | 34.20±1.57 | 1.27±0.10 | 108.25±4.67 |
| *A. pasteurianus*/pT-aal | 0.67±0.06 | 0.16±0.00 | 0.94 | 38.86±1.80 | 1.47±0.09 | 80.75±6.82 |
| **Overexpression of cofactor PQQ module** | | | | | | |
| *A. pasteurianus*/pT-pqqAB | 0.67±0.08 | 0.15±0.01 | 0.85 | 29.93±1.95 | 1.30±0.07 | 187.00±2.97 |
| *A. pasteurianus*/pT-pqqABCDE | 0.73±0.04 | 0.13±0.00 | 0.75 | 30.00±1.50 | 1.18±0.05 | 1181.25±7.36 |
| **Combinational overexpression of dehydrogenases and cofactor** | | | | | | |
| *A. pasteurianus*/pT-adhA-pqqAB | 0.74±0.02 | 0.22±0.01 | 1.28 | 35.44±0.44 | 1.42±0.01 | 117.50±4.78 |
| *A. pasteurianus*/pT-adhA-pqqABCDE | 0.79±0.02 | 0.23±0.01 | 1.33 | 38.01±0.45 | 1.54±0.04 | 128.50±5.23 |
| *A. pasteurianus*/pT-aldh-pqqAB | 0.71±0.04 | 0.21±0.01 | 1.20 | 37.05±0.37 | 1.49±0.08 | 133.75±3.95 |
| *A. pasteurianus*/pT-aldh-pqqABCDE | 0.67±0.02 | 0.19±0.01 | 1.09 | 36.57±0.83 | 1.45±0.07 | 196.25±6.54 |
| *A. pasteurianus*/pT-aal-pqqAB | 0.65±0.08 | 0.16±0.01 | 0.91 | 39.14±1.58 | 1.30±0.09 | 98.00±5.91 |
| *A. pasteurianus*/pT-aal-pqqABCDE | 0.56±0.01 | 0.10±0.00 | 0.59 | 35.05±0.57 | 0.92±0.07 | 95.72±3.20 |

**Table S3.** Summary of growth and production characteristics of *A. pasteurianus* B7003 and recombinants in fermentation medium (containing 4% (v/v) ethanol) supplemented with 1% (v/v) initial acetic acid. (The acetic acid yield has been subtracted from the initial acetic acid)

| Strains | OD_600_ | Growth rate  (h^-1^) | Relative fitness (W) | Acetic acid yield (g·L^-1^) | Acetic acid productivity  (g·L^-1^·h^-1^) | PQQ concentration (μg·L^-1^) |
| --- | --- | --- | --- | --- | --- | --- |
| *A. pasteurianus* B7003 | 0.47±0.03 | 0.13±0.01 | 1.00 | 32.22±1.38 | 1.30±0.01 | 17.95±0.34 |
| *A. pasteurianus*/pBBR1MCS-2 | 0.51±0.03 | 0.13±0.01 | 1.01 | 32.55±0.50 | 1.25±0.03 | 16.92±0.82 |
| **Overexpression of dehydrogenase module** | | | | | | |
| *A. pasteurianus*/pT-adhA | 0.34±0.05 | 0.12±0.00 | 0.88 | 32.35±1.45 | 1.19±0.01 | 20.01±0.81 |
| *A. pasteurianus*/pT-aldh | 0.41±0.03 | 0.11±0.00 | 0.82 | 35.28±2.21 | 1.26±0.05 | 22.22±0.77 |
| *A. pasteurianus*/pT-aal | 0.42±0.03 | 0.11±0.01 | 0.85 | 41.21±0.83 | 1.61±0.07 | 19.05±0.90 |
| **Overexpression of cofactor PQQ module** | | | | | | |
| *A. pasteurianus*/pT-pqqAB | 0.24±0.02 | 0.06±0.00 | 0.47 | 36.84±0.55 | 1.02±0.04 | 36.19±1.57 |
| *A. pasteurianus*/pT-pqqABCDE | 0.27±0.05 | 0.05±0.00 | 0.40 | 35.23±1.22 | 0.96±0.04 | 95.35±0.08 |
| **Combinational overexpression of dehydrogenases and cofactor** | | | | | | |
| *A. pasteurianus*/pT-adhA-pqqAB | 0.49±0.03 | 0.14±0.00 | 1.08 | 35.77±1.19 | 1.37±0.04 | 19.13±0.69 |
| *A. pasteurianus*/pT-adhA-pqqABCDE | 0.53±0.04 | 0.16±0.00 | 1.21 | 37.50±0.84 | 1.48±0.02 | 33.26±1.66 |
| *A. pasteurianus*/pT-aldh-pqqAB | 0.49±0.03 | 0.14±0.00 | 1.08 | 35.86±1.81 | 1.35±0.00 | 19.65±0.91 |
| *A. pasteurianus*/pT-aldh-pqqABCDE | 0.38±0.02 | 0.10±0.00 | 0.78 | 35.80±1.00 | 1.39±0.02 | 29.39±1.34 |
| *A. pasteurianus*/pT-aal-pqqAB | 0.53±0.04 | 0.13±0.00 | 0.99 | 41.75±0.95 | 1.65±0.09 | 25.08±1.13 |
| *A. pasteurianus*/pT-aal-pqqABCDE | 0.53±0.02 | 0.14±0.00 | 1.08 | 41.69±0.82 | 1.67±0.04 | 51.56±0.18 |

**Table S4.** Summary of growth and production characteristics of *A. pasteurianus* B7003 and recombinants in fermentation medium (containing 4% (v/v) ethanol) supplemented with 3% (v/v) initial acetic acid. (The acetic acid yield has been subtracted from the initial acetic acid)

| Strains | OD_600_ | Growth rate  (h^-1^) | Relative fitness (W) | Acetic acid yield (g·L^-1^) | Acetic acid productivity  (g·L^-1^·h^-1^) | PQQ concentration (μg·L^-1^) |
| --- | --- | --- | --- | --- | --- | --- |
| *A. pasteurianus* B7003 | 0.17±0.01 | 0.02±0.00 | 1.00 | 32.98±0.94 | 0.65±0.02 | 8.86±0.23 |
| *A. pasteurianus*/pBBR1MCS-2 | 0.12±0.02 | 0.01±0.00 | 0.65 | 31.79±1.57 | 0.65±0.08 | 11.77±0.53 |
| **Overexpression of dehydrogenase module** | | | | | | |
| *A. pasteurianus*/pT-adhA | 0.13±0.01 | 0.01±0.00 | 0.55 | 28.35±1.35 | 0.43±0.09 | 9.62±0.42 |
| *A. pasteurianus*/pT-aldh | 0.13±0.01 | 0.01±0.00 | 0.65 | 28.45±1.40 | 0.43±0.10 | 8.78±0.37 |
| *A. pasteurianus*/pT-aal | 0.13±0.00 | 0.01±0.00 | 0.45 | 25.20±0.58 | 0.21±0.10 | 7.19±0.37 |
| **Overexpression of cofactor PQQ module** | | | | | | |
| *A. pasteurianus*/pT-pqqAB | 0.13±0.01 | 0.01±0.00 | 0.70 | 26.40±1.63 | 0.33±0.07 | 12.49±0.59 |
| *A. pasteurianus*/pT-pqqABCDE | 0.15±0.01 | 0.02±0.00 | 0.80 | 28.32±1.05 | 0.52±0.05 | 25.50±0.73 |
| **Combinational overexpression of dehydrogenases and cofactor** | | | | | | |
| *A. pasteurianus*/pT-adhA-pqqAB | 0.25±0.03 | 0.04±0.00 | 1.90 | 28.22±0.93 | 0.55±0.01 | 10.60±0.35 |
| *A. pasteurianus*/pT-adhA-pqqABCDE | 0.24±0.02 | 0.03±0.01 | 1.55 | 29.44±1.94 | 0.56±0.04 | 11.74±0.52 |
| *A. pasteurianus*/pT-aldh-pqqAB | 0.20±0.01 | 0.02±0.00 | 1.15 | 25.43±2.21 | 0.31±0.08 | 13.45±0.63 |
| *A. pasteurianus*/pT-aldh-pqqABCDE | 0.16±0.02 | 0.01±0.00 | 0.70 | 24.60±0.42 | 0.28±0.07 | 8.77±0.44 |
| *A. pasteurianus*/pT-aal-pqqAB | 0.23±0.03 | 0.03±0.00 | 1.50 | 25.82±1.95 | 0.32±0.10 | 10.60±0.35 |
| *A. pasteurianus*/pT-aal-pqqABCDE | 0.22±0.09 | 0.02±0.01 | 1.00 | 28.56±1.71 | 0.23±0.03 | 4.39±0.27 |


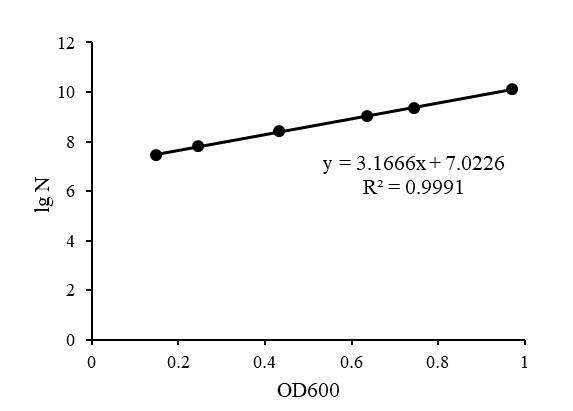


**Fig. S1.** The relationship between the OD_600_ and the number of living bacteria (N, CFU/mL) in *A.pasteurianus* (N = 10^3.1666x+7.0226^).


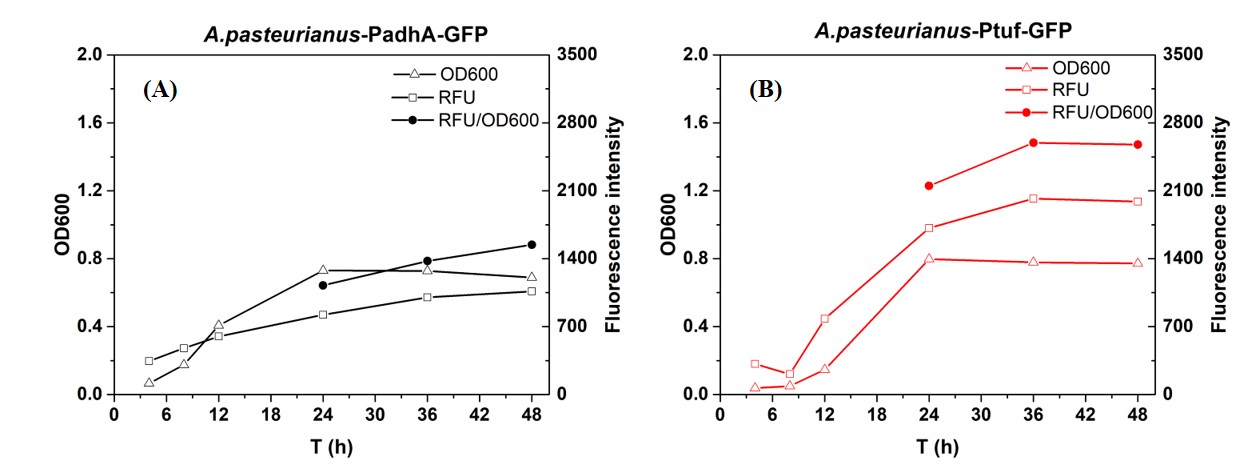


**Fig. S2.** The whole cell fluorescence intensities of strains with P_adhA_-GFP (A) and P_tuf_ -GFP (B). The promoter strength was defined as RFU/OD_600_ (relative fluorescence unit divided by the corresponding OD_600_)


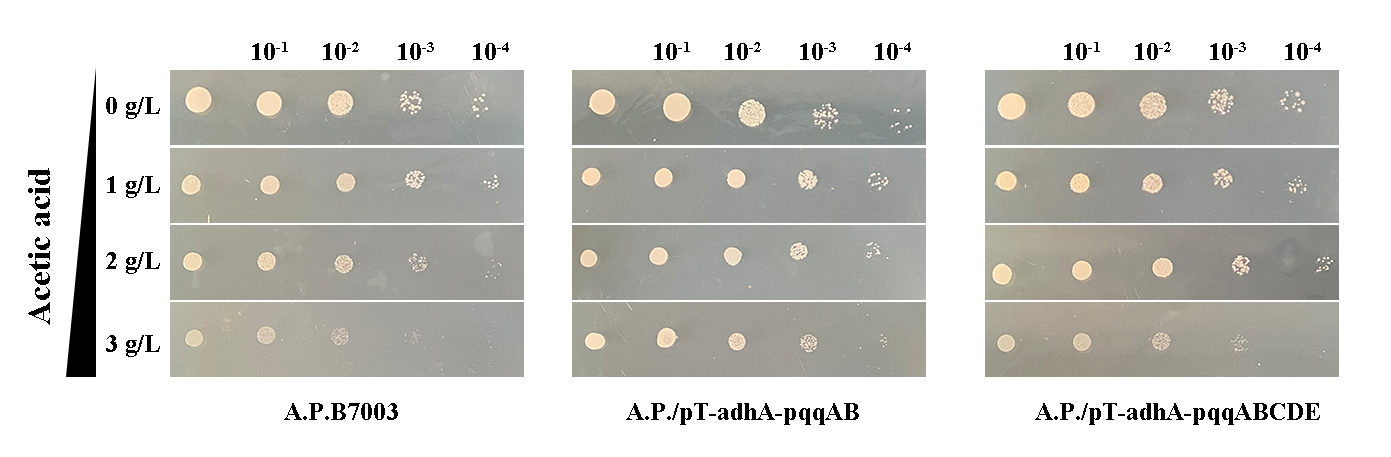


**Fig.S3.** Acetic acid tolerance of wild-type strain *A. pasteurianus* B7003, *A. pasteurianus*/pT-adhA-pqqAB and *A. pasteurianus*/pT-adhA-pqqABCDE. Cells were spotted in serial dilutions (diluted by a factor 10) and grown on fermentation medium agar plates amended with various concentrations of acetic acid.
